# Radiomic predicts early response to CDK4/6 inhibitors in hormone receptor positive metastatic breast cancer

**DOI:** 10.1038/s41523-023-00574-7

**Published:** 2023-08-11

**Authors:** Mohammadhadi Khorrami, Vidya Sakar Viswanathan, Priyanka Reddy, Nathaniel Braman, Siddharth Kunte, Amit Gupta, Jame Abraham, Alberto J. Montero, Anant Madabhushi

**Affiliations:** 1 Department of Biomedical Engineering, Emory University, Atlanta, GA USA; 2 Department of Medicine, Division of Hematology and Oncology, University Hospitals/Seidman Cancer Center, Case Western Reserve University, Cleveland, OH USA; 3 Department of Biomedical Engineering, Case Western Reserve University, Cleveland, OH USA; 4 Taussig Cancer Institute, Cleveland Clinic, Cleveland, OH USA; 5 Atlanta VA Medical Center, Atlanta, GA USA

**Keywords:** Predictive markers, Prognostic markers

## Abstract

The combination of Cyclin-dependent kinase 4/6 inhibitors (CDK4/6i) and endocrine therapy (ET) is the standard of care for hormone receptor-positive (HR + ), human epidermal growth factor receptor 2-negative (HER2-) metastatic breast cancer (MBC). Currently, there are no robust biomarkers that can predict response to CDK4/6i, and it is not clear which patients benefit from this therapy. Since MBC patients with liver metastases have a poorer prognosis, developing predictive biomarkers that could identify patients likely to respond to CDK4/6i is clinically important. Here we show the ability of imaging texture biomarkers before and a few cycles after CDK4/6i therapy, to predict early response and overall survival (OS) on 73 MBC patients with known liver metastases who received palbociclib plus ET from two sites. The delta radiomic model was associated with OS in validation set (HR: 2.4; 95% CI, 1.06–5.6; *P* = 0.035; C-index = 0.77). Compared to RECIST response, delta radiomic features predicted response with area under the curve (AUC) = 0.72, 95% confidence interval (CI) 0.67–0.88. Our study revealed that radiomics features can predict a lack of response earlier than standard anatomic/RECIST 1.1 assessment and warrants further study and clinical validation.

## Introduction

Endocrine therapy (ET) is highly effective in the treatment of hormone receptor-positive (HR + ), and human epidermal growth factor receptor 2-negative (HER2-) metastatic breast cancer (MBC) and currently is the preferred first line treatment^1,2^. However, about half of the patients who receive ET will eventually develop therapeutic resistance within 1–2 years, and subsequently derive limited clinical benefit^3^.

More recently, the addition of cyclin-dependent kinase 4/6 inhibitors (CDK4/6i) to ET have been shown to significantly delay the development of therapeutic resistance^4^, and consequently, in several phase 3 randomized trials have significantly improved progression-free survival (PFS) compared to ET alone among patients with advanced HR + , HER2-negative breast cancer^5–7^. Moreover, data from MONALEESA-2^8^, MONALEESA-3^9^, and MONALEESA-7^10^ trials have shown significant improvement in median overall survival (OS) with the addition of CDK4/6i to ET.

CDK4/6i effectively blocks the cell cycle proliferation from G1 (pre-DNA synthesis) to the S phase (DNA synthesis) by blocking the CDK4/6-cyclin D1 complex and preventing cancer cell proliferation and treatment resistance^4^. Thus far, considerable effort has been made to identify predictive and prognostic biomarkers for CDK4/6i across all phase 3 randomized trials, including PALOMA, MONALEESA, and MONARCH^11–13^.

The identification of predictive biomarkers that can reliably identify which HR + , HER2- MBC patients will clinically benefit from CDK4/6i therapy has been challenging. Recently published studies performing analysis from tissue samples from the PALOMA-2 trial explored several biomarkers including genomic loss of the CDK4/6 inhibitor p16, Cyclin D1 amplification, or complete loss of Rb (the target of CDK4/6 action). Unfortunately, none of these biomarkers showed clear promise, except for the rare tumor with complete Rb loss, which as expected, was resistant to CDK4/6i.^14,15^. Consequently, despite several phase 3 trials demonstrating the benefit of the addition of CDK4/6i to ET in either first or second-line metastatic settings, there are no predictive biomarkers that can identify patients likely to benefit from CDK4/6i^16^.

The duration of therapy on endocrine therapy and CDK4/6i can vary rather dramatically depending on the site of metastatic disease. Patients with ER + MBC and bone-only disease have a much more favorable prognosis than those with visceral metastases—approximately 33% are progression-free on CDK4/6i and first-line endocrine therapy at 60 months^17^.

By contrast, the presence of liver metastases in patients with ER + MBC portends a very poor prognosis with an estimated median OS of only approximately 2 years^18,19^, even with CDK4/6i plus ET^20^. In PALOMA-3, the liver was the most common site for visceral metastases, affecting 62.5% of the population with PFS of 7.5 months in patients treated with ET plus CDK4/6i than with ET alone (2.4). In addition, the median PFS was significantly longer in patients treated with ET plus CDK4/6i than with placebo plus ET in the presence of visceral metastases (9.2 months versus 3.4 months). In PALOMA-2, liver metastases were present in 35.0% of the population with PFS of 13.7 versus 8.4 months. The median PFS in PALOMA-2 in patients with visceral metastases was significantly longer in those treated with CDK4/6i plus letrozole compared with letrozole alone (19.3 months versus 12.9 months). Consequently, there is an urgent unmet clinical need for novel predictive biomarkers that can rationally guide the use of CDK4/6i to identify patients most likely to benefit from treatment and novel prognostic biomarker to identify patient’s overall survival—particularly in those with liver or visceral metastases that have a much shorter median OS than patients with bone-only disease—avoid time on ineffective medications, as well as mitigate financial toxicities and potential adverse effects (neutropenia, diarrhea, transaminase elevation, diarrhea) in those unlikely to respond. This will allow oncologists to adjust treatment options early on and develop more successful therapeutic strategies to overcome endocrine resistance in CDK4/6i non-responders^21^.

Recently, computerized feature analysis of radiographic scans or radiomic analysis has demonstrated significant potential for response prediction to chemo- and targeted therapy in breast cancer^22,23^. These radiomic approaches can computationally capture quantitative measurements of tumor heterogeneity and its microenvironment in radiological images, such as computed tomography (CT). In the metastatic setting, patients usually undergo serial CT scans throughout treatment to monitor disease progression. Recent evidence shows that CDK4/6i enhances tumor antigen presentation during therapy^24^. These micro-architectural changes in the tumor might precede changes in radiographic features during CDK4/6i treatment.

In this study, we utilized a delta radiomic-based analysis of breast cancer patients with liver metastases on pre- and a few cycles post-treatment CT to predict early treatment response to CDK4/6i therapy. We hypothesized that quantitative capture of textural changes of the lesion and the surrounding microenvironment in the CT scan before and a few cycles after treatment in women with liver metastases on CDK4/6i therapy may provide a better accurate characterization of treatment response compared to the textural pattern on CT scan before initiating therapy. Towards this end, we used CT scans from 73 patients with ER + /HER2- MBC and the presence of liver metastasis at baseline- and a few cycles post-treatment with CDK4/6i. We sought to identify radiomic features associated with RECIST response and OS in ER + /HER2- MBC patients treated with CDK4/6i by interrogating the tumor and tumor microenvironment on CT imaging.

## Results

Of the 32 patients from University Hospitals/Seidman Cancer Center (S_t_), 65% of patients had an objective response or stable disease and 35% had progressive disease on ET and CDK4/6 therapy at the date of the last follow-up. The median age at diagnosis was 63 years [35–82]. In total 21 of them were White, 4 were African American, and race information was unavailable for the remaining 7 patients. 65% of patients received palbociclib as 1st or 2nd line therapy and the remaining received a different CDK4/6i, i.e., ribociclib or abemaciclib. A total of 5/32 of the patients were treated with CDK4/6 inhibitors as 1^st^ line and the remaining 27/32 patients as 2^nd^ line therapy. After initiating ET/CDK4/6i therapy, 24/32 (75%) of the patients had a progression of the disease. The median time from the start date of CDK4/6i to the date of progression was 12 months (95% CI, 7.6–16.5), and the median date of the last follow-up was 16 months (95% CI, 9–22.8). In addition, the median OS for the patients in S_t_ was 18.15 months (95% CI, 11.26–25).

Of the 41 patients from Cleveland Clinic (S_v_), at the date of the last follow-up, 22 had an objective response or stable disease and 19 had progressive disease. The median age at diagnosis was 58 [36–79] years. Out of the 41 patients, 16 were White, 3 were African American, and the self-reported race information for the remaining patients was not available. The median OS for the patients in S_v_ was 19.43 months (95% CI, 14.93–23.93).

A univariable Cox regression analysis identified that OS did not significantly differ for: race (White vs. African American) (hazard ratio, HR: 0.85 (95% CI, 0.22–3.26); *P* = 0.81; Concordance Index, C-index = 0.54), age (HR: 1 (95% CI, 0.95–1.04); *P* = 0.99; C-index = 0.48), or tumor volume (before CDK4/6i/ET therapy) (HR: 1.2; 95% CI, 0.71–2; *P* = 0.49; C-index = 0.56).

### Delta Radiomic features from pre- and post-treatment CT scans were associated with OS in patients treated with CDK4/6i

**Fig. 1:**
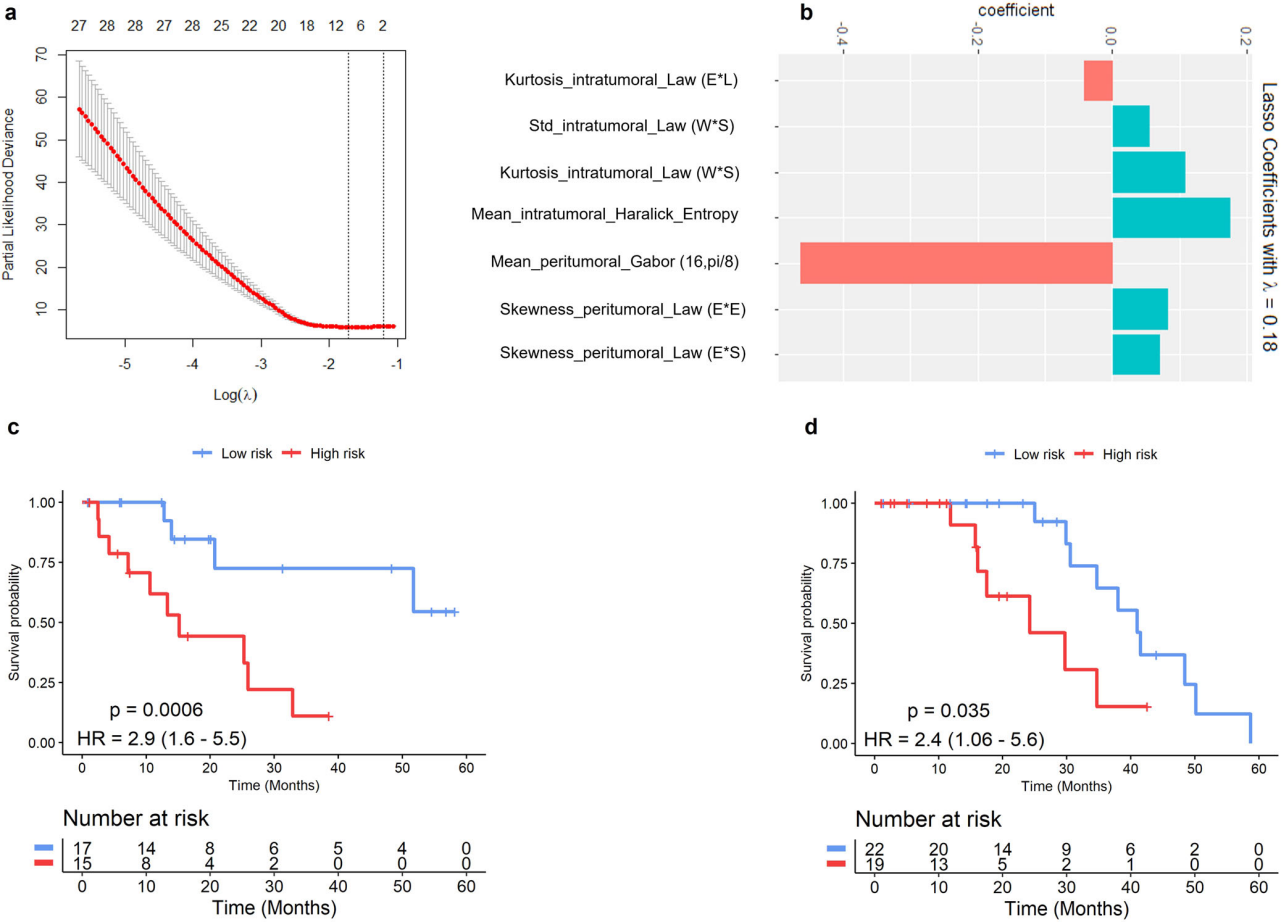
Radiomic risk score and its association with OS. **a** The lambda value of 0.18 minimizes Mean Squared Error (MSE) within 100-fold cross validation in training set. **b** Most prognostic radiomic features with their corresponding coefficients. **c** Kaplan–Meier survival curves for the patients in the training set. **d** Kaplan–Meier survival curves for the patients in the validation set. A significant association of the radiomic risk score with the OS was shown in the training and validation sets. A log-rank test was employed to compare survival curves between two groups.

Within S_t_, 7 radiomic features were obtained from 1110 radiomic features after feature pruning from the LASSO model. The LASSO model selected 7 radiomic features with a lambda value of 0.18 (see Fig. 1a). Details of the selected features and their coefficients have been listed in Fig. 1b. Of the 7 radiomic features, 3 were picked from the peritumoral region and 4 were selected from the intra-tumoral region. The RRS ranged from −1.58 to 2.87 and the optimum cut-off value (the median) was found to be −0.119. Based on this value, patients were divided into high- and low-risk groups. A univariable Cox regression analysis developed using radiomic features indicated that RRS was significantly associated with OS in S_t_ (HR: 2.9 (95% CI, 1.6–5.5); *P* = 0.0006; C-index = 0.82) and S_v_ (HR: 2.4 (95% CI, 1.06–5.6); *P* = 0.035; C-index = 0.77). Median survival time in high and low-risk groups was 12.58 and 23.17 months, respectively (*P* = 5.7e-04). In a multivariable analysis using a combination of clinical and radiomic features, the RRS alone was found to be significantly associated with OS in S_t_ (risk-score: HR = 2.65 (95% CI: 1.47–4.8), *P* = 0.0012; age: HR = 1, 95% CI: 0.95–1.06, *P* = 0.8; race: HR = 0.56, 95% CI: 0.07–4.2, *P* = 0.57; baseline tumor vol: HR = 1.8 (95% CI: 0.72–4.4), *P* = 0.2; C-index = 0.83) and S_v_ (risk-score: HR = 2.4 (95% CI: 1.02–5.6), *P* = 0.044; tumor vol: HR = 1.2 (95% CI: 0.78–1.86), *P* = 0.4; C-index = 0.78). The corresponding Kaplan–Meier survival curves showed a significant difference in OS between patients with low and high RRS both in S_t_ and S_v_ (*P* < 0.05). Kaplan–Meier survival curves for S_t_ and S_v_ are shown in Fig. 1c, d, respectively.

**Fig. 2:**
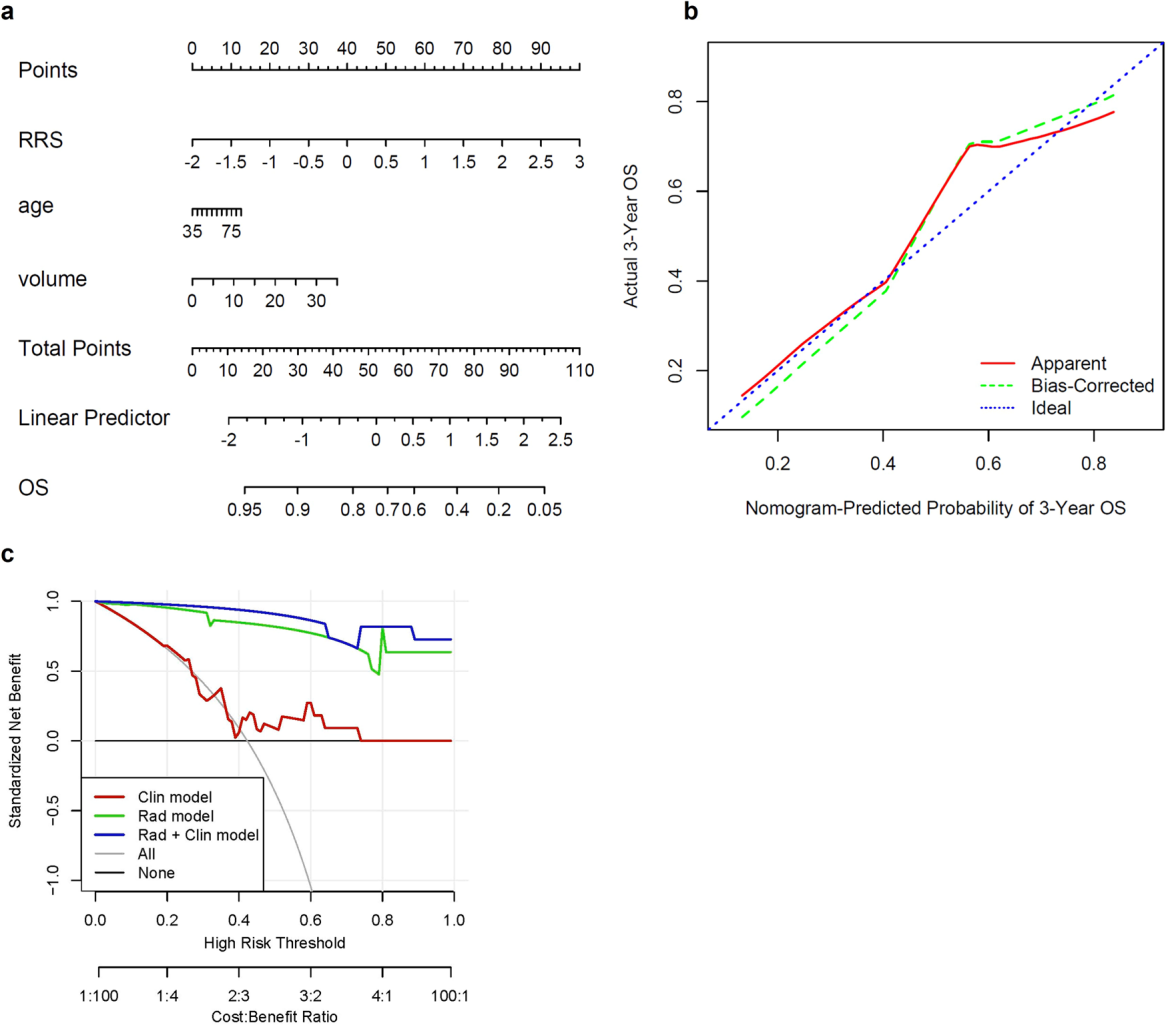
Radiomics nomogram model and calibration curve for predicting survival probability. **a** A nomogram that quantifies the probability of 3-year survival in ER + MBC patients treated with CDK4/6i plus ET. **b** Calibration curve for survival. The blue dotted line shows an ideal agreement between actual and predicted probabilities of survival. Dots correspond to apparent predictive accuracy. **c** Decision curve analysis (DCA) for three models (clinical, radiomic, and integrated radiomic+clinical). The integrated model has the highest net benefit in predicting which high-risk patients should receive more aggressive treatment, as compared with radiomic model and a clinical model alone; and simple strategies such as treating all patients or no patients.

A radiomics nomogram model incorporating the radiomics signature with clinical biomarkers was the model that best predicted OS with a C-index of 0.83 (95% CI, 0.73–0.91) in S_t_ compared to the clinical or radiomics model alone (Fig. 2a). In S_v_, the C-index was 0.79 (95% CI, 0.71–0.86). The clinical model alone had a lower prognostic performance (compared to radiomics alone) in predicting OS with a C-index of 0.57 (95% CI, 0.40–0.75) in S_t_.

The calibration plot (Fig. 2b) demonstrated a good fit between nomogram-predicted and observed OS. The Hosmer-Lemeshow test yielded a *p*-value of 0.47, suggesting no significant difference between predicted and observed OS. The DCA was used to demonstrate the clinical decision utility of the nomogram. Figure 2c shows DCA for three models (clinical model, radiomic model, and integrated clinical plus radiomics model). The integrated model had the highest net benefit in the prediction of high-risk patients (those with poor OS) to receive more intensive treatment (e.g., chemotherapy) than the clinical model or radiomics model alone.

### Tumor volumetric changes during CDK4/6i therapy were not associated with OS

The median tumor volume in S_t_ was 2.57 mL (range, 0.23–33.32 mL) before CDK4/6i administration and 3.9 mL (range, 0.121–20.84 mL) after therapy. The tumor change size during therapy was not statistically associated with OS, neither in S_t_ (HR: 0.82 (95% CI, 0.43–1.58); *P* = 0.56; C-index = 0.46), nor in S_v_ (HR: 0.7 (95% CI, 0.34–1.42); *P* = 0.32; C-index = 0.49).

### Delta radiomic features predict response to ET/CDK4/6i therapy

**Fig. 3:**
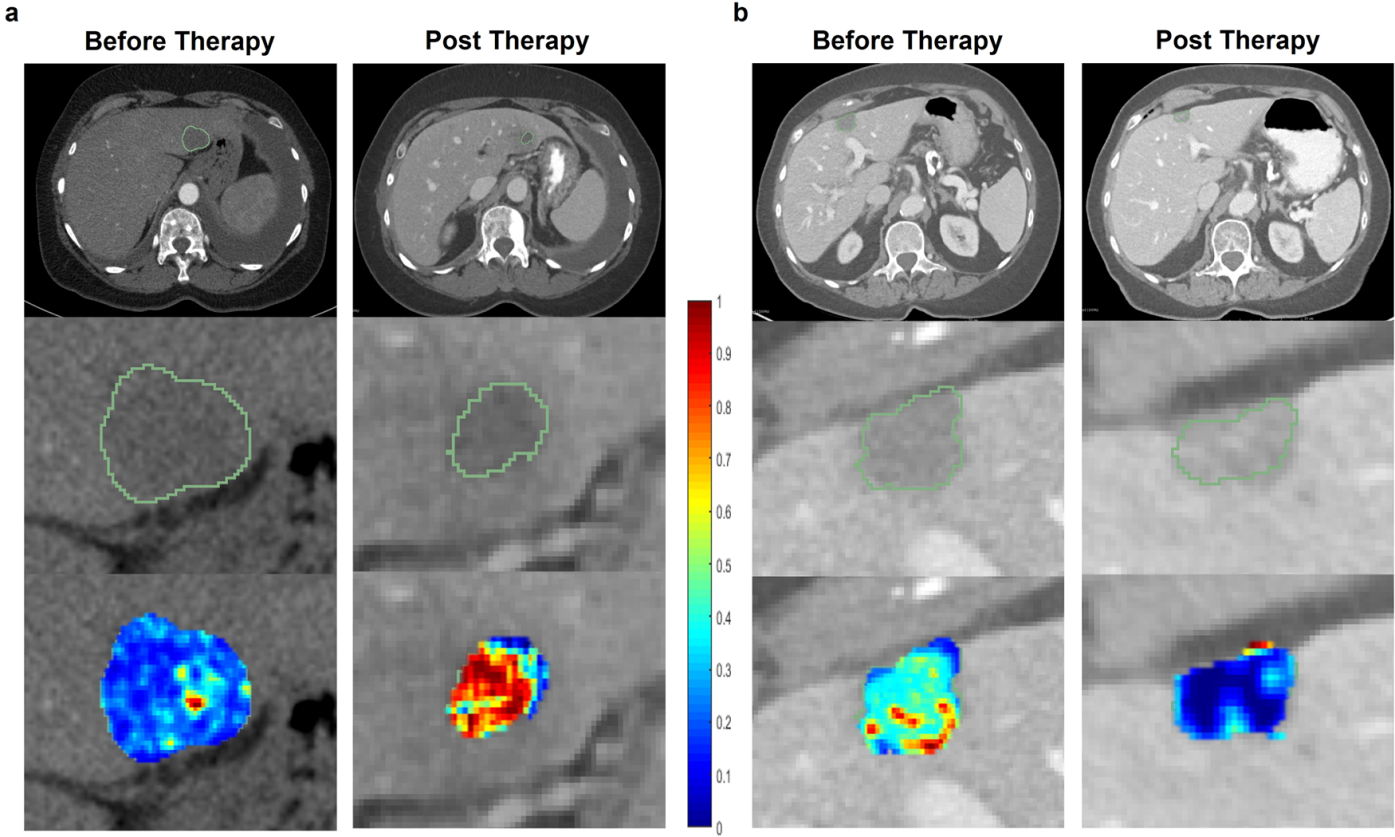
Delta radiomic features predict response to CDK4/6i therapy. Axial contrast enhanced CT images (top row), liver tumor segmentations (middle row), and heatmaps (lower row) of intra-tumoral Haralick (entropy) feature in the representative pre- and post-treatment CT scans of a non-responder **a** and a responder **b**. The time between pre- and post-treatment CT scans for the non-responder was 2.1 months and for the responder was 1.9 months. The images depict an elevated expression of Haralick entropy post-therapy in the non-responder as compared to the responder (bottom row in **a**, **b**).

Figure 3a, b illustrate the change of the intratumoral Haralick entropy feature for representative non-responder and responder patients before and a few cycles after CDK4/6i therapy. We observed an elevated expression of Haralick entropy post-therapy in the non-responders as compared to the responders. Moreover, the LDA classifier trained with identified prognostic features yielded an AUC of 0.74 (95% CI, 0.61–0.98) on S_t_. Prediction accuracy was slightly different between patients with thin slice thickness (1 mm) and thick slice thickness (3 and 5 mm), but it was not statistically significant (AUC on thin slice thickness was 0.75 vs. 0.73 on thick slice thickness, *P* > 0.05). Within S_v_, the classifier yielded an AUC of 0.72 (95% CI, 0.67–0.88), with an accuracy of 0.7, sensitivity of 0.67, and specificity of 0.86 for response prediction.

### Comparison of delta radiomics with baseline radiomic features

An LDA classifier trained with a combination of 7 baseline radiomic feature in S_t_ yielded an AUC of 0.7 (95% CI, 0.65–0.77) as compared to delta radiomics (AUC 0.74; *P* = 0.02) and corresponding AUCs of 0.69 on S_v_, respectively. The risk score generated by baseline texture features was associated with OS in S_t_ (HR: 2.1(95% CI, 1.23–4.4); *P* = 0.005; C-index = 0.73) and S_v_ (HR: 1.98 (95% CI, 1.01–3.16); *P* = 0.046; C-index = 0.69).

## Discussion

The use of CDK4/6i with ET has revolutionized the management of HR + HER2- metastatic breast cancer due to their favorable toxicity profiles and their relevant antitumor activity^5,7,25^. The US Food and Drug Administration (FDA) and European Medicines Agency (EMA) have approved the clinical use of CDK4/6i such as palbociclib, ribociclib, and abemaciclib in metastatic breast cancer patients with HR + /HER2- in the first line setting as well as the second line in combination with ET. In clinical trials, all FDA approved CDK4/6i when combined with ET have demonstrated a significant prolongation of PFS compared with ET alone^4,7^.

Approximately half of all patients with metastatic breast cancer develop liver metastases and 5–12% of patients exhibit liver metastases as the primary site of breast cancer recurrence^26,27^. If untreated, liver metastases are associated with a dismal prognosis and even with treatment with ET and CDK4/6i the median OS is only 24 months^18,19,28^. In addition, ER + MBC patients with visceral metastases compared to patients with the bone predominant disease have a significantly shorter median PFS to ET ± CDK4/6i^17,29^. Hence, there is an urgent need for clinically useful predictive biomarkers that can identify patients with liver metastases likely to benefit from ET/CDK4/6i.

Many previous studies have investigated the role of molecular alterations in the tumor such as pRB, RB1 mutations, CCDN1amplifications, and CCNE1 overexpression as potential biomarkers for CDK4/6i response but these investigations have not yielded any identification of a clinically useful predictive biomarker so far^15,30,31^. A growing body of research suggests that loss of the retinoblastoma tumor suppressor gene (Rb), leads to accelerated angiogenesis and tumor progression which is one of the most important biomarkers associated with acquired resistance and lower PFS to CDK4/6i^32,33^.

In this study, we investigated the role of delta radiomic features on the baseline and the first ET and CDK4/6i treatment assessment CT scan in HR + /HER2- MBC patients with liver metastasis to predict response and OS. From a practical perspective, the development of a radiomic score that could identify patients less likely to respond to CDK4/6i therapy based on baseline CT would be of interest with regard to adoption in clinical practice. In this study, we showed that feature variations before and a few cycles after therapy can predict response even more accurately compared to baseline CT images. A nomogram model that integrated radiomic scores with clinical biomarkers was developed in this study. Our nomogram model showed that radiomic scores had a better prognostic performance for predicting OS compared to clinical biomarkers alone. Moreover, the decision curve analysis (DCA) showed that the radiomic score had a better overall net benefit compared to clinical biomarkers for predicting high-risk patients suitable to receive more aggressive therapy across several threshold probability values. Age was included in our nomogram, as it has previously been shown to be an independent adverse prognostic factor in women with metastatic breast cancer and liver metastases^18^.

To the best of our knowledge, this work is the first study that has explored radiomic feature analysis to predict the response of ET plus CDK4/6i in ER + MBC patients as well as overall prognosis.

We found that higher intratumoral Haralick entropy that captures tumor heterogeneity was associated with non-response to CDK4/6i/ET and poor OS. It is important to acknowledge that both the training and validation cohorts have a median OS of approximately 2 years which is much shorter than what is observed in patients with bone-only disease, which represents a different biology, as well as the reported overall OS in all phase 3 CDK4/6 trials. As previously discussed, a median OS of 24 months is consistent with the published literature in ER + MBC with liver metastases. Our selection of a very poor prognosis subset of ER + MBC was intentional and represents a limitation of the study.

Previous studies have shown that tumor heterogeneity increment is indicative of genomic heterogeneity and is associated with a worse prognosis in non-small cell lung cancer patients treated with immunotherapy or chemotherapy^34–36^. By contrast, decrement in intratumoral heterogeneity is associated with a favorable response to therapy and prolonged PFS. A previous study by Wander et al. showed that genomic alterations in RB1, AURKA, and CCNE2 expression enhance resistance to CDK4/6i therapy. In other words, heterogeneity may be a radiomic feature that is likely correlated with increased resistance to CDK4/6i therapy^33^. While entropy has not directly been compared to genomic clonal evaluation, it could potentially help better define tumor heterogeneity.

Angiogenesis is another hallmark of cancer proliferation and tumor metastases^37^. Architectural disorder of the tumor-associated vascular network has recently been shown to be a marker of therapeutic response to several treatment strategies in breast cancer^38^. We found that peritumoral Laws texture increase during therapy may capture angiogenesis and tumor microenvironment heterogeneity increase, which is associated with poor therapeutic response and OS^32,33,39^.

In addition, p16 (tumor suppressor) downregulation leads to increased HIF-α which in turn causes tumor hypoxia, which is known to confer therapeutic resistance^40^. Prior evidence suggests that a hypoxic tumor environment might be captured by radiomic texture analysis of lesions extracted from CT images^34^.

Moreover, there is also some evidence suggesting that CDK4/6i can elicit their therapeutic response by enhancing the activation of T-cells^41^. It can be postulated that peri-tumoral Gabor texture increase during therapy might be capturing the presence of immune T-cells around the tumor, caused by CDK4/6i therapy and might be an indicator of better response to therapy.

We have identified a new imaging-based biomarker to monitor response in patients undergoing CDK4/6i therapy. The ability to determine response during a few cycles of treatment will allow early adjustment of treatment regimens. In the future, such validated image-based radiomic biomarkers can potentially identify non-responders and will enable oncologists to predict residual endocrine sensitivity and reduce ineffective treatment, toxicity, and side effects associated with CDK4/6i therapy and timely change to other effective target therapies, including subsequent CDK4/6 and PI3K/AKT/mTOR inhibitors^42^. Such validated biomarkers can also identify those patients that would benefit from CDK4/6i versus those patients that would benefit from ET.

We acknowledge that our study has several limitations. While we used two independent cohorts of patients for building and validating our model, the cohort size in this study is relatively small but it is quite challenging to assemble large cohorts for this problem due to a small number of patients treated with this relatively new therapy. The second limitation is the retrospective nature of our study, not a prospective study. To tackle this limitation, validation on a large multi-site prospective cohort is required. Also, there are questions on variability in scanning differences between scanners such as convolution kernels, reconstruction algorithms, and slice thickness, that hinder the widespread applicability of radiomic features, although some studies have shown novel radiomic features that are relatively immune to differences in image-related variabilities^43,44^. Also, further work needs to be done to perform extensive stratified analyses to explore the relationship between the molecular and mutational status of the tumors and radiomics in this patient population. Moreover, we need more prospective studies with multiparametric evaluation, including known prognostic factors such as performance status and time to relapse to develop and validate the signature as a prognostic biomarker.

We hope to address these limitations in future work. In addition, we need to develop and validate this signature as predictive of the maximum benefit of CDK4/6i therapy vs. ET alone. However, this will require a prospective trial design to determine the ability of the radiomic signature to predict benefit to ET plus CDK4/6i therapy.

Nonetheless, despite these limitations, our study revealed that dynamic change of CT-based radiomic texture features between baseline and a few cycles post-treatment of HR + , HER2- breast cancer patients with liver metastasis can predict early response and OS to CDK4/6i coupled with ET.

## Methods

### Study population

This multi-institutional study included HR+ metastatic breast cancer (MBC) patients with liver metastasis, who received palbociclib (palbo), ribociclib (ribo), or abemaciclib (abema) as first- or second-line therapy in combination with ET.

Patient with HR + MBC, with liver metastasis and available baseline and post-treatment CT abdomen/pelvis at University Hospitals/Seidman Cancer Center (UHSCC, *n* = 52) and Cleveland Clinic (CCF, *n* = 45) were identified from prospective registries of patients with ER + /HER2- MBC with overall clinical outcomes which has previously been published^45^. The median time between pre- and post-treatment CT was 3.5 months (95% CI, 3.2–3.88).

Scans of patients not suitable for feature extraction, such as those with CT scan artifacts and poor image quality, absence of post-treatment scans, or non-contrast CTs were excluded. This resulted in a total of *n* = 32 patients from UHSCC and *n* = 41 patients from CCF. UHSCC was used for training (S_t_) and CCF was used as an independent validation cohort (S_v_).

The study conformed to Health Insurance Portability and Accountability Act (HIPAA) guidelines and was approved by the Institutional Review Board (IRB) at University Hospitals (STUDY20201206) and Cleveland Clinic (IRB 19–559). The IRB waived the requirements for patient informed consent due to the retrospective and observational nature of this study.

### Clinical endpoints

The primary endpoint of this study was OS, defined as the time from the date of ET and CDK4/6i initiation to the recorded date of death, or censored at the last known date of follow-up. The secondary endpoint was response status at the date of the last follow-up, as defined by RECIST v1.1. Patients who had progressive disease were classified as non-responders and patients who had a complete response, partial response, or stable disease were classified as responders.

### Lesion segmentation and feature extraction

Baseline and post-treatment CTs were acquired either on Siemens, GE Medical Systems, Philips, or Toshiba scanners according to standard scanning protocol at CCF and UHSCC institutions. The imaging protocol included a tube voltage of 100 to 120 kVp, slice thickness ranging from 1 to 3 mm, and standard convolution kernel reconstruction. All patients were injected with a contrast agent before imaging. An expert reader reviewed and annotated liver lesions on baseline and post-treatment scans using a freehand tool on 3D-Slicer® software^46^. The annotations were verified by a board-certified radiologist (14 years of experience). The primary lesion—noted in the radiology report and corresponding to the largest post-treatment lesion—was chosen as the region of interest for radiomic analysis. If liver lesions could not be confidently defined on CT scans, a recent dedicated liver MRI was utilized for confirmation of metastasis. Radiomic texture features were extracted on a pixel level from pre-treatment and post-treatment CT scans. For each scan, a set of intra- and peritumoral radiomic features were considered. The peritumoral rim around the lesion was defined via the use of quantitative morphological operations (dilation) as a region extending radially from the lesion boundary up to roughly 12 mm around the tumor. The choice of peritumoral compartment rim size was defined based on the previously described method^47^, including the normal appearing liver parenchyma surrounding the metastatic lesion and excluding regions containing peri-hepatic fat (fat has much lower attenuation compared to normal liver parenchyma at CT, with a range of −10 to −100 HU)^48^.

**Fig. 4:**
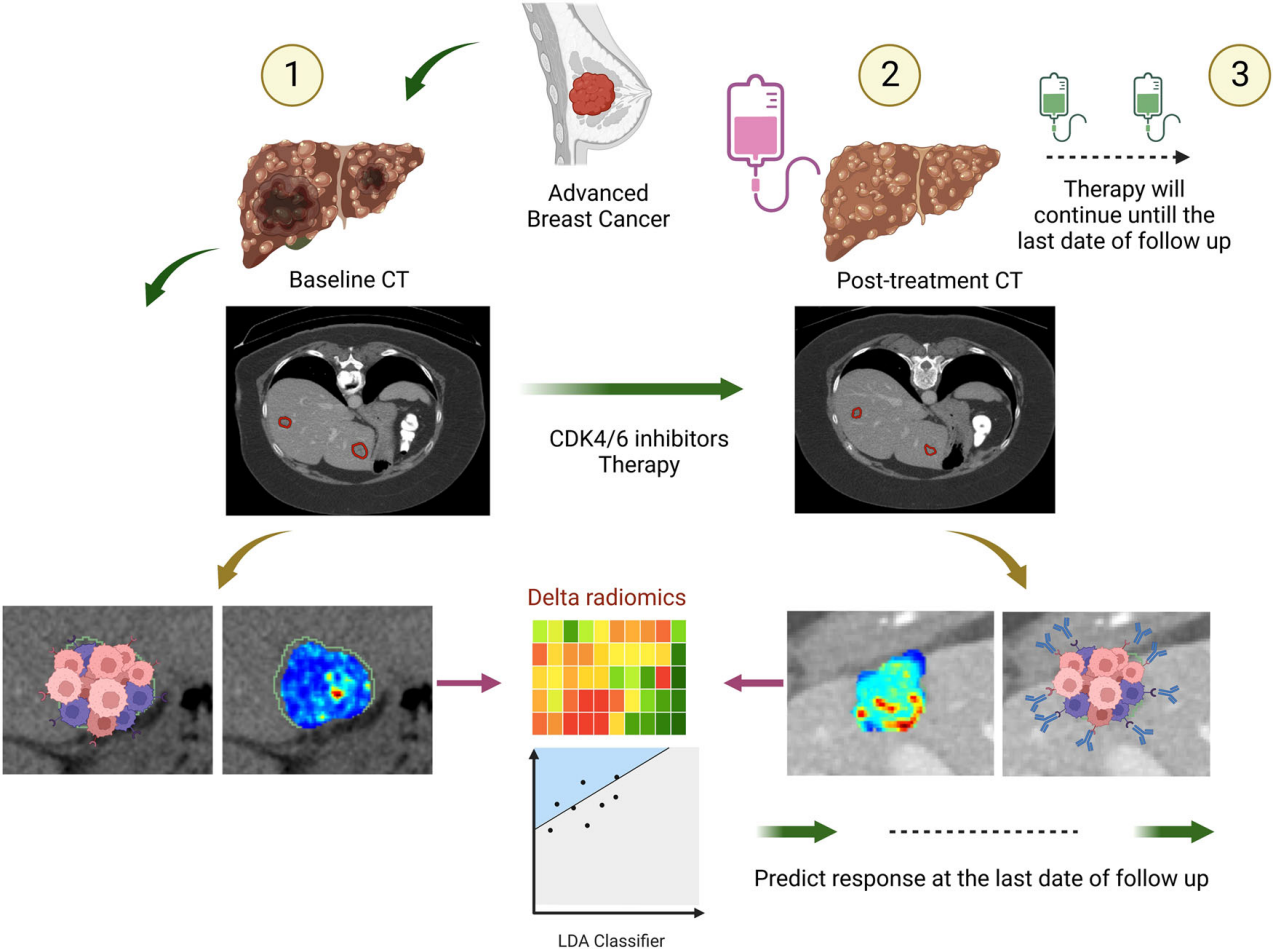
Overall experimental design. The CT images of metastatic breast cancer patients before starting therapy were acquired. After a few cycles of therapy, post-therapy images were collected. From both the baseline and post-therapy CTs, radiomic features were extracted, and delta radiomic features were calculated. Subsequently, a machine learning classifier was trained using the delta radiomic features to predict the response at the end of the therapy cycle. This figure was created with Biorender.com.

Radiomic texture features (consisting of 13 Haralick texture features^49^, 25 Laws features^50^, 25 Laws Laplacian (smoothing image with Laplacian filter and then extract Laws feature), and 48 Gabor features^51^) were extracted from 2D contours, in a slice-by-slice manner across all annotated slices of the lesion, on both pre-treatment and post-treatment scans. These features capture textural patterns, heterogeneity, and local appearance of the tumor and its microenvironment on CT and reflect hallmarks of tumor biology. Each texture feature was summarized by five first-order statistics (mean, median, SD, skewness, kurtosis) separately within the tumor and peritumoral region, resulting in a total of 555 texture feature statistics. Moreover, 24 shape features that capture aspects of the 3D lesion structure including the size, volume, and longest diameter of the tumor were also extracted. All radiomic feature values were then normalized (mean = 0 and SD = 1) and the change of feature statistics between baseline and post-treatment scans was calculated to yield the feature set. The overall experimental design for this study is shown in Fig. 4.

### Statistical analysis

The least absolute shrinkage and selection operator (LASSO) method was used to select the most prognostic features to OS in S_t_^52^. The top selected features along with their corresponding coefficients were used for radiomic risk score (RRS) construction. RRS was calculated via a linear combination of selected features with corresponding coefficients. The value of the tuning parameter (λ) in LASSO was selected based on a grid search of λ on 100-fold cross-validations in a manner to minimize Mean Squared Error (MSE) within each fold. The LASSO Cox regression model was performed using the “glmnet” package in R.

A risk score threshold was chosen in S_t_ to stratify patients into high and low-risk groups based on the median of RRS. The prognostic performance of RRS was validated using Kaplan-Meier survival analysis, log-rank test, HR, and Harrell’s concordance index (C index). Univariate analysis of RRS and the clinical–pathological variables was performed to evaluate the association of each marker with OS. Multivariable Cox regression analysis was used to investigate the independent prognostic value of the RRS relative to clinical-pathological variables.

A prognostic nomogram was also developed on S_t_ by combining clinical and prognostic radiomic features and validated on S_v_. To evaluate nomogram performance, C indices were calculated from the nomogram for RRS, clinical factors alone, and RRS plus clinical factors. The calibration plot for the nomogram was evaluated by reviewing the plots of nomogram-predicted survival probabilities with Kaplan-Meier estimated probabilities along with the Hosmer-Lemeshow test, a statistical test for goodness of fit for logistic regression models. A *p*-value < 0.05 implies that the model is not a good fit whereas the converse suggests that there is no evidence of poor fit. Bootstraps with 500 resamples were employed to quantify model overfitting and for calculating Kaplan-Meier estimates. The nomogram model was generated by the use of R with the “rms” package (Regression Modeling Strategies). A decision curve analysis (DCA) was performed to evaluate the clinical efficacy of the radiomics model by assessing the net benefits of the model across a range of threshold probabilities^53^.

Additionally, a linear discriminant analysis (LDA) classifier was used to evaluate the ability of identified prognostic features to predict response to therapy. The LDA classifier generates linear class boundaries (i.e., linear patterns) while assuming that the covariance of each class is identical. The classifier performance for predicting response was evaluated by the area under the receiver operating characteristic (ROC) curve (AUC). In S_t_, the classifier performance was assessed by averaging the AUC values computed over 100 iterations of threefold cross-validation (CV). The trained classifier was eventually tested for response prediction within S_v_.

Finally, any differences between clinical categories were assessed using Fisher’s exact test, where a two-sided t-test was used for continuous variables. A bilateral *P*-value < 0.05 was considered statistically significant.

### Supplementary information

nr-reporting-summary

## Data Availability

Data are available upon reasonable request. Access to datasets from the Cleveland Clinic and the University Hospitals Cleveland Medical Center (used with permission for this study) should be requested directly from these institutions via their data access request forms. Subject to the institutional review boards’ ethical approval, unidentified data would be made available as a test subset. All experiments and implementation details are described thoroughly in the Materials and methods section so they can be independently replicated with non-proprietary libraries.

## Code availability

Details and codes for feature extraction, feature selection and statistical analysis are available at https://github.com/Hadi-Khorrami.
