# Supplementary material for: Radiomic predicts early response to CDK4/6 inhibitors in hormone receptor positive metastatic breast cancer
Source: NPJ Breast Cancer. 2023 Aug 11;9:67. doi: 10.1038/s41523-023-00574-7 (PMC10421862; doi:10.1038/s41523-023-00574-7)
Supplement: Supplementary file 1 — nr-reporting-summary [file 41523_2023_574_MOESM1_ESM.pdf]

nature portfolio

Corresponding author(s): Anant Madabhushi

Last updated by author(s): Jul 25, 2023

Reporting Summary

Nature Portfolio wishes to improve the reproducibility of the work that we publish. This form provides structure for consistency and transparency in reporting. For further information on Nature Portfolio policies, see our Editorial Policies and the Editorial Policy Checklist.

Statistics

For all statistical analyses, confirm that the following items are present in the figure legend, table legend, main text, or Methods section.

n/a	Confirmed	
☐	☒	The exact sample size (*n*) for each experimental group/condition, given as a discrete number and unit of measurement
☐	☒	A statement on whether measurements were taken from distinct samples or whether the same sample was measured repeatedly
☐	☒	The statistical test(s) used AND whether they are one- or two-sided<br>*Only common tests should be described solely by name; describe more complex techniques in the Methods section.*
☐	☒	A description of all covariates tested
☐	☒	A description of any assumptions or corrections, such as tests of normality and adjustment for multiple comparisons
☐	☒	A full description of the statistical parameters including central tendency (e.g. means) or other basic estimates (e.g. regression coefficient) AND variation (e.g. standard deviation) or associated estimates of uncertainty (e.g. confidence intervals)
☐	☒	For null hypothesis testing, the test statistic (e.g. *F*, *t*, *r*) with confidence intervals, effect sizes, degrees of freedom and *P* value noted<br>*Give P values as exact values whenever suitable.*
☒	☐	For Bayesian analysis, information on the choice of priors and Markov chain Monte Carlo settings
☒	☐	For hierarchical and complex designs, identification of the appropriate level for tests and full reporting of outcomes
☒	☐	Estimates of effect sizes (e.g. Cohen's *d*, Pearson's *r*), indicating how they were calculated

Our web collection on statistics for biologists contains articles on many of the points above.

Software and code

Policy information about availability of computer code

Data collection	No software was used
Data analysis	Matlab 2019, R 3.6.3, Python

For manuscripts utilizing custom algorithms or software that are central to the research but not yet described in published literature, software must be made available to editors and reviewers. We strongly encourage code deposition in a community repository (e.g. GitHub). See the Nature Portfolio guidelines for submitting code & software for further information.

Data

Policy information about availability of data

All manuscripts must include a data availability statement. This statement should provide the following information, where applicable:

- Accession codes, unique identifiers, or web links for publicly available datasets
- A description of any restrictions on data availability
- For clinical datasets or third party data, please ensure that the statement adheres to our policy

Data are available upon reasonable request. Access to datasets from the Cleveland Clinic and the University Hospitals Cleveland Medical Center (used with permission for this study) should be requested directly from these institutions via their data access request forms. Subject to the institutional review boards' ethical approval, unidentified data would be made available as a test subset. All experiments and implementation details are described thoroughly in the Materials and

methods section so they can be independently replicated with non-proprietary libraries. Details and codes for feature extraction, feature selection and statistical analysis are available at https://github.com/Hadi-Khorrami.

Human research participants

Policy information about studies involving human research participants and Sex and Gender in Research.

Reporting on sex and gender	73 Women with HR+ metastatic breast cancer (MBC) with liver metastasis were included in this study
Population characteristics	Of the 32 patients from University Hospitals/Seidman Cancer Center (St), 65% of patients had objective response or stable disease and 35% had progressive disease at the date of the last follow-up. The median age at diagnosis was 63 years [35 – 82]. 21 of them were White, 4 were African American, and race information was unavailable for the remaining 7 patients. 65% of patients received palbociclib as 1st or 2nd line therapy and the remaining received a different CDK4/6i, i.e., Ribociclib or Abemaciclib. 5/32 of the patients were treated with CDK4/6 inhibitors as 1st line and the remaining 27/32 patients as 2nd line therapy. Of the 41 patients from Cleveland Clinic (Sv), at the date of the last follow-up, 22 had an objective response or stable disease and 19 had progressive disease. The median age at diagnosis was 58 [36 – 79] years. Out of the 41 patients, 16 were White, 3 were African American, and the self-reported race information for the remaining patients was not available.
Recruitment	A chart review was performed to identify patients with HR+ MBC, with liver metastasis and available baseline and post-treatment CT abdomen/pelvis at University Hospitals/Seidman Cancer Center (UHSCC, n=52) and Cleveland Clinic (CCF, n=45).
Ethics oversight	The study conformed to Health Insurance Portability and Accountability Act (HIPAA) guidelines and was approved by the Institutional Review Board (IRB) at University Hospitals (STUDY20201206) and Cleveland Clinic (IRB 19-559). The IRB waived the requirements for patient informed consent due to the retrospective and observational nature of this study.

Note that full information on the approval of the study protocol must also be provided in the manuscript.

Field-specific reporting

Please select the one below that is the best fit for your research. If you are not sure, read the appropriate sections before making your selection.

☒ Life sciences ☐ Behavioural & social sciences ☐ Ecological, evolutionary & environmental sciences

For a reference copy of the document with all sections, see nature.com/documents/nr-reporting-summary-flat.pdf

Life sciences study design

All studies must disclose on these points even when the disclosure is negative.

Sample size	A chart review was performed to identify patients with HR+ MBC, with liver metastasis and available baseline and post-treatment CT abdomen/pelvis at University Hospitals/Seidman Cancer Center (UHSCC, n=52) and Cleveland Clinic (CCF, n=45).
Data exclusions	Scans of patients not suitable for feature extraction, such as those with CT scan artifacts and poor image quality, absence of post-treatment scans, or non-contrast CTs were excluded. This resulted in a total of n=32 patients from UHSCC and n=41 patients from CCF.
Replication	We independently validated our constructed predictive and prognostic model from training on the validation set to evaluate the robustness of the built models.
Randomization	Our study does not involve random allocation of patient samples into different experimental groups. The patients from one institute was used for training and patients from other institute was used for validation.
Blinding	The investigators were blinded to the to group allocation during data collection but the investigators had aware of patient's outcome and it was not blinded validation study

Reporting for specific materials, systems and methods

We require information from authors about some types of materials, experimental systems and methods used in many studies. Here, indicate whether each material, system or method listed is relevant to your study. If you are not sure if a list item applies to your research, read the appropriate section before selecting a response.

Materials & experimental systems

n/a	Involved in the study
☒	☐ Antibodies
☒	☐ Eukaryotic cell lines
☒	☐ Palaeontology and archaeology
☒	☐ Animals and other organisms
☒	☐ Clinical data
☒	☐ Dual use research of concern

Methods

n/a	Involved in the study
☒	☐ ChIP-seq
☒	☐ Flow cytometry
☒	☐ MRI-based neuroimaging
